# Spontaneous Cure of a Nævus Maternus—Large Vascular Tumor Occupying Side of Neck

**Published:** 1894-09

**Authors:** A. Bethune Patterson

**Affiliations:** Atlanta, Ga.; 204 Equitable Building


### SPONTANEOUS CURE OF A NÆVUS MATERNUS—LARGE VASCULAR TUMOR OCCUPYING SIDE OF NECK.

BY A. BETHUNE PATTERSON, M.D., ATLANTA, GA.

In May, 1878, I was called to see an infant daughter of Mr. and Mrs. T., of Barnwell, S. C. She was healthy and well-developed. A few days after birth the nurse noticed a growth about the size of a small pea back of the right ear, situated in the angle formed by ramus of inferior maxillary and mastoid process. Some anxiety had arisen in the minds of the parents, as the growth was increasing in dimensions, and as I did not understand its nature advised non-interference. At two months old the tumor had reached the size of a plum, globular, smooth and elastic; skin not adherent; no pulsation, and bore none of the signs of inflammation. At this stage a neighboring physician was consulted; thinking he had to deal with something that would bear the use of the knife, lanced it; blood flowed freely, and some trouble experienced in arresting it. A week or two subsequently I was sent for and urged by parents, who thought the doctor had not gone deep enough, to reach the pus they believed existed. I tried to be sure to not fall into the supposed error of my colleague, and sent my lancet deep into the growth, and was somewhat chagrined at not seeing pus flow. My mortification did not stop here, for it really looked as if the child would bleed to death before I could stop the flow. I observed no decrease in the bulk from the hemorrhage. From this date the tumor grew rapidly. At four months of age measured eight inches in circumference. The tumor grew tense under mental emotions. At the age of one year it occupied nearly the entire side of the face. Lobe of ear pressed up at right angles with plane of face and encroaching upon the eye, partially closing palpebral fissure, extending down the neck to clavicle. It looked immense, greatly circumscribing the movements of the head and neck. At one and a half years it had reached its greatest dimensions, and remained apparently stationary, when, at the age of two years, it began to decrease, and at four years had quite left the face, and appeared as a pendulous, flabby mass, occupying the side of the neck. The character of the tumor could now be distinctly made out. The pendulous body was a mass of blood vessels about the size of a whip-cord, forming a globular tumor about the size of a man's fist. Absorption went on gradually, when at the age of ten years all that could be felt was a thick mass about the size of an almond and a pendulous fold of skin overlying it, just back of the ear. At twelve years nothing could be felt of the growth. The mother states that she was advised by physicians to guard against falls and blows, as such accidents were likely to result in rupture and death from hemorrhage. Thus the possibility of these vessels having been occluded from these causes might be reasonably excluded. Gross says: "A spontaneous cure occasionally occurs; the vessels become obliterated, possibly as an effect of inflammation leading to the coagulation of their contents and the gradual closure of their caliber." I am unable to advance any theory to explain the phenomena which bears the imprint of physiological law.

In October, 1893, I met the subject of this report, now Miss L. M. T., in infancy a hideous deformity, carrying with it the unceasing solicitude of a distressed mother for her unfortunate child, and the sympathy of all who saw it. She is now a tall, handsome brunette, the pride of her mother's heart, and the belle of the community, with nothing to denote the site of the growth, except a slight depression in angle formed by mastoid and ramus, in which are to be seen, on very close inspection, two short white lines ever pointing to the blunders of two country doctors. May it prove a warning to others to exercise caution in the use of the lancet in doubtful conditions.

204 *Equitable Building.*

---

MANAGEMENT OF SYCOSIS.—Clip the beard very close, when an excellent soothing application is Lassar's paste, made as follows: Starch and zinc oxide, each two drachms; salicylic acid, fifteen grains; vaseline, one ounce. Before application the crust should be removed by soaking with oil and washing with soap and water. —*Ex.*

